# Adolescent-Led Mental Health Promotion in the IMPACT Programme: Vignette Development with Indigenous Youth

**DOI:** 10.1177/21501319261448309

**Published:** 2026-08-13

**Authors:** Paulo T. C. Jardim, Antonio José Grande, Ieda Avila Vargas Dias, Maria Gabriela Curubeto Godoy, Xanthi Zourntos, Divya Parmar, Jamie Murdoch, Marcia Andrea Soardi, Andreia Jacomini, Nelio Campos, Seeromanie Harding

**Affiliations:** 1 School of Medicine, 67708 State University of Mato Grosso do Sul, Mato Grosso do Sul, Campo Grande, Brazil; 2 28113 School of Medicine, Federal University of Juiz de Fora, Juiz de Fora, Brazil; 3 28124 School of Medicine, Federal University of Rio Grande do Sul, Rio Grande do Sul, Porto Alegre, Brazil; 4 Department of Population Health Sciences, School of Population Health & Environmental Sciences, Faculty of Life Sciences & Medicine, 4616 King’s College London, London, UK; 5 Department of Health and Education, Rio Grande do Sul, Porto Alegre, Brazil

**Keywords:** Mental health, Indigenous adolescence, Kaingang, Health promotion, Vignettes, Interculturality

## Abstract

**Introduction:**

Indigenous adolescents experience unique biopsychosocial challenges compounded by cultural disruption, territorial displacement, and systematic marginalization. These factors contribute to elevated rates of psychological distress and suicide among Indigenous adolescent, necessitating culturally responsive mental health programmes that center youth voice and cultural values.

**Objective:**

To develop and evaluate adolescent-led art-based vignettes as a culturally sensitive methodology for promoting mental health discussions among Kaingang Indigenous adolescents in the IMPACT study (Implementation of a multi-sectoral programme to improve Indigenous adolescent mental health in Brazil and Dominica)

**Method:**

We conducted an exploratory qualitative study using participatory action research principles. Twenty-four Kaingang adolescents (ages 10-18) from two Indigenous schools participated in 12 workshops over six months. Participants created vignettes across multiple artistic formats (videos, drawings, songs) addressing school, family, and community contexts. Data collection included 10 group dynamics with adolescents, teachers, health professionals, and community leaders. Thematic analysis following Braun and Clarke’s framework was conducted with cultural validation from community members.

**Results:**

Participants created 29 vignettes addressing key themes including cultural discrimination, substance use, environmental degradation, family dynamics, and spiritual practices. Protective factors identified included family support, sports participation, and community connections. Risk factors encompassed increased substance use, family conflict, discrimination, and environmental destruction. Vignettes effectively facilitated dialogue about sensitive topics including psychological distress, cultural identity, and belonging while creating safe spaces for collective reflection.

**Conclusion:**

Adolescent-led vignette creation represents a promising, culturally grounded approach to Indigenous adolescent mental health promotion. This methodology successfully fostered meaningful dialogue, adolescent agency, and reflective engagement while honoring cultural values and community knowledge systems. Despite limitations in sample size, findings support expanding this participatory approach across diverse Indigenous contexts to strengthen intercultural mental health programmes.

## Introduction

Adolescence represents a critical developmental period characterized by profound biopsychosocial transformations and identity formation processes. For Indigenous youth, this transition occurs within distinctive cultural frameworks shaped by ancestral connections, territorial relationships, and collective worldviews that differ fundamentally from Western developmental paradigms.^1^ Unlike conventional Western conceptualizations of prolonged adolescence, most Indigenous communities recognize rapid transitions from childhood to adulthood mediated by biological maturation, ceremonial rites, and community-defined social roles.^2^ This cultural specificity underscores the importance of understanding Indigenous adolescent development within its proper sociocultural context.

A country in South America Indigenous population comprises approximately 1.6 million individuals representing over 300 distinct ethnic groups, each maintaining unique cultural practices, languages, and territorial connections.^3^ This remarkable diversity profoundly influences how Indigenous adolescents navigate identity construction, community belonging, and future aspirations. However, these developmental processes increasingly occur against a backdrop of systematic marginalization, cultural disruption, and limited opportunities, contributing to psychological distress and uncertainty about future prospects.^4^

The psychological wellbeing of Indigenous adolescents faces unprecedented challenges rooted in historical trauma and ongoing structural inequities. Feelings of discouragement and disconnection often stem from territorial displacement, suppression of traditional knowledge systems, and inadequate living conditions—factors that extend beyond individual experiences to reflect broader patterns of cultural erasure, institutional racism, and rights violations.^5^ These collective traumas manifest in alarming mental health outcomes, with suicide rates among Indigenous populations rising from 9.3 per 100,000 in the early 2000s to 17.6 per 100,000 by 2020. Notably, 64% of these deaths occur among individuals under 24 years of age, with the Amazon region and Mato Grosso do Sul accounting for the majority of cases.^6,7^

The Pan American Health Organization emphasizes that Indigenous suicide must be understood as manifestation of collective suffering encompassing territorial, cultural, spiritual, and political dimensions rather than isolated individual acts.^8^ This perspective is supported by epidemiological data revealing distinct patterns of self-harm among Indigenous populations, including higher rates of poisoning incidents concentrated in younger age groups (1-4 years and 18-19 years) compared to the general population.^9^

Substance use presents additional challenges within Indigenous communities. While alcohol traditionally served ceremonial and cultural functions, such as fermented cassava beverages, increasing urbanization and village integration into broader municipal areas has facilitated access to commercial alcohol products. Combined with social marginalization and weakening cultural boundaries, these factors contribute to disproportionate alcoholism rates of 17.6% among Indigenous populations compared to 5-6% among non-Indigenous, often leading to increased aggressive behaviors and additional mental health complications.^10,11^

Despite recognition of these critical needs, Indigenous health subsystem lacks adequate mental health infrastructure. Primary care teams typically exclude psychologists and psychiatrists, who remain concentrated in urban centers often distant from Indigenous territories. Mental health care falls predominantly to nurses, nursing technicians, and general practitioners with minimal specialized training in Indigenous-specific cultural competencies or mental health programmes.^12,13^

This gap between need and available resources necessitates innovative, culturally grounded approaches to Indigenous adolescent mental health promotion. Effective programmes must strengthen community connections, facilitate meaningful dialogue, and honor Indigenous life projects and worldviews while building local capacity for sustainable mental health support.

The IMPACT study (Implementation of a Multisectoral Program to Improve the Mental Health of Indigenous Adolescents), funded by the Global Alliance for Chronic Diseases, represents one such innovative approach. Conducted with Kaingang and Guarani adolescents Indigenous Territory, this initiative employs artistic methodologies to promote mental health discussions while incorporating Indigenous perspectives and strengthening educational and health system responses to adolescent needs.

Central to the IMPACT programme is a series of 12 adolescent-led workshops involving Indigenous school students, health professionals, and community leaders. These sessions utilize vignettes, participant-created written or audiovisual materials representing specific subjects, moments, or realities from the creator’s perspective,^14^ as pedagogical tools for facilitating dialogue about emotions, conflicts, belonging, and wellbeing within culturally appropriate contexts. This article describes the collaborative vignette creation process, co-authored with participating adolescents and teachers, demonstrating how youth-led artistic expression can serve as a foundation for culturally responsive mental health promotion.

## Methods

### Study Design

This qualitative study employed an exploratory, participatory action research design centered on collaborative vignette creation as a culturally responsive methodology for examining Indigenous adolescent mental health perspectives.

### Ethics Approval and Consent to Participate

The study received ethical approval from the Research Ethics Committee of the Federal University of Juiz de Fora (Opinion No. 5,966) and adhered to all ethical principles governing research with human participants and Indigenous communities. All methods were performed in accordance with the relevant guidelines and regulations of the National Health Council (Resolution No. 466/12) and Indigenous research protocols.

Informed consent was obtained from all participants and/or their legal guardians prior to participation in the study. For adolescents aged 15–18 years, informed consent was obtained directly from the participants. For minors aged 10–14 years, informed consent was obtained from a parent and/or legal guardian, and assent was also obtained from the adolescents themselves. All research activities respected Indigenous community protocols and were conducted with the approval of local leaders and school representatives from the Kaingang communities.

### Theoretical Framework and Rationale

The study’s theoretical foundation integrated community-based participatory research principles with Indigenous knowledge systems to explore mental health contexts, cultural values, and lived experiences among Indigenous adolescents. Given the cultural sensitivity required for mental health promotion in Indigenous contexts, we employed collaborative vignette construction as our primary methodological approach. Vignettes served as symbolic devices designed to facilitate identification, reflection, and knowledge exchange while enabling dialogue about conflicts, school experiences, spirituality, relationships, and emotional wellbeing.

Vignettes represent a well-established qualitative methodology particularly suited for cross-cultural research contexts where researcher influence must be minimised and participant voice maximised.^14^ This approach proved especially relevant within Indigenous cultural frameworks, where silence, respect, and listening carry meanings that differ from those typically associated with dominant Western models of communication and engagement.

### Setting and Context

The study was conducted in Guarita Indigenous Territory, involving students from two local Indigenous schools in elementary and secondary education levels within the Kaingang community.

### Participants and Recruitment

Participants were recruited through individual meetings in which the study objectives were explained and informed consent was obtained, in accordance with Indigenous community protocols. Eligibility criteria included: (i) expressed interest in participating in the study; (ii) satisfactory academic standing; (iii) prior engagement in school-based activities; and (iv) availability to attend the scheduled workshops.

To enhance representativeness, recruitment sought to reflect internal community diversity, ensuring balanced inclusion across gender, age groups, and social roles.

A total of 24 Indigenous adolescents were enrolled and allocated into two developmentally appropriate groups: 12 participants aged 10–14 years and 12 participants aged 15–18 years. All participants were enrolled in local Indigenous schools and represented diverse family and community backgrounds within the territory.

Given the eligibility criteria, there is potential for selection bias toward adolescents who are more engaged in school settings. Consequently, youth experiencing greater academic challenges, social withdrawal, or more severe psychological distress may be underrepresented. This limitation is further addressed in the Study Limitations section.

### Vignette development protocol

Phase 1: Initial Training (August 2023) Researchers conducted four one-hour training sessions introducing participants to vignette concepts and applications as communication tools. Participants were organized into two groups and learned that vignettes could encompass multiple creative formats including written narratives, comic strips, podcasts, videos, songs, poems, drawings, photographs, digital art, parodies, and dance performances.

As a practical exercise, adolescents worked in small groups to create initial mental health-focused vignettes, which were subsequently presented to a larger audience including teachers, researchers, health professionals, and community leaders. Post-presentation discussions addressed creation process advantages and challenges, with researchers providing facilitation strategies to support ongoing production.

Phase 2: Vignette Creation and Discussion (September-November 2023) Data collection occurred through ten bi-weekly group sessions, each lasting approximately 90 minutes. Group dynamics based on active methodologies facilitated group interactions around specific mental health topics, with participants creating vignettes across three domains: school, family, and community contexts. These vignettes illustrated cultural values, lived experiences, protective factors, and challenges related to mental health preservation.

#### Each Session Followed a Structured Three-Phase Format

##### Individual Creation Phase

Participants independently developed vignettes representing their daily experiences and perspectives.

##### Group Presentation Phase

Creators shared their vignettes with the larger group.

##### Collective Discussion Phase

Guided discussions explored vignette meanings using standardized prompts:

“What do you consider beneficial for mental health and why?”

“What do you view as harmful to mental health and why?”

“How do these representations correspond to your experiences?”

“What types of support are needed and why?”

“What key mental health promotion messages can vignettes convey?”

Vignette production increased progressively across sessions as participants developed comfort and motivation with the creative process. Each meeting incorporated discussion of previously created vignettes alongside generation of new content.

Phase 3: Final Assessment The tenth session provided comprehensive evaluation of activities and outcomes across the development period. Field notes were maintained throughout all phases to ensure protocol fidelity and document creative processes.

### Data Analysis

All data underwent thematic analysis following Braun and Clarke’s^15^ six-phase framework: data familiarization, initial coding, theme searching, theme reviewing, theme defining and naming, and report production. The analytical team comprised four researchers: two Indigenous community health researchers embedded in the Kaingang territory and two external academic researchers with expertise in qualitative health research and Indigenous mental health. In the data familiarization phase, all four researchers independently reviewed transcripts, field notes, and vignette content. Initial coding was conducted independently by two researchers (one community researcher and one academic researcher), after which codes were compared and reconciled in joint sessions attended by all four team members. Theme generation and review were conducted collaboratively across all four researchers, with community researchers providing continuous cultural validation of emerging interpretations. Analysis proceeded with active participation from teachers and community representatives to ensure cultural validation of all interpretations.

Given the multimodal nature of the data, all vignette formats—including videos, drawings, songs, and other artistic expressions—were systematically incorporated into the analysis. Audiovisual materials were transcribed or descriptively summarized, while visual content was interpreted through detailed field notes capturing symbolic elements, narratives, and contextual meanings. These materials were treated as qualitative data and coded alongside focus group transcripts, allowing for integrated thematic development across modalities.

Methodological rigor followed Standards for Reporting Qualitative Research (SRQR) guidelines. Data triangulation incorporated multiple sources (focus group transcripts, field notes, vignette content, participant feedback) to corroborate findings and enhance interpretation reliability. Researcher reflexivity was continuously practiced to identify and mitigate potential cultural biases in data collection and analysis processes.

Member checking involved inviting participants to review final interpretations, ensuring result validation and maintaining community ownership of findings. This collaborative approach honored Indigenous research principles emphasizing community control over knowledge production and dissemination.

### Researcher Positionality

The research team comprised both Indigenous and non-Indigenous researchers, including community-based health researchers embedded within the Kaingang territory and external academic investigators. This composition enabled continuous dialogue between insider and outsider perspectives throughout the research process. Reflexivity was actively practiced to acknowledge potential power imbalances and cultural biases, with Indigenous researchers playing a central role in guiding data interpretation and ensuring cultural validity. Engagement with community members, teachers, and local leaders further supported accountability and alignment with Indigenous research principles.

## Results

### Overview of Vignette Production

Participants created 29 vignettes across multiple artistic formats including videos, drawings, photographs, posters, songs, parodies, and comic strips. Elementary school participants (ages 10-14) produced 16 vignettes, while high school participants (ages 15-18) created 13 vignettes. Content analysis revealed five major thematic domains that emerged consistently across age groups and creative formats: cultural identity and practices, environmental degradation, prejudice and discrimination, violence and substance use, and social integration and resistance. Distribution of Vignettes by Educational Level and Domain is presented in Table 1.

**Table 1.** Distribution of Vignettes by Educational Level and Domain

Educational level	School domain	Family domain	Community domain	Total
Elementary	4	5	8	16
High School	5	3	5	13
Total	9	8	13	29

## Cultural Identity and Practices

### Traditional Crafts as Cultural Expression

Crafts emerged as a central theme representing cultural continuity and identity preservation across all three domains. Participants demonstrated sophisticated understanding of craftsmanship as embodying ancestral knowledge and cultural values. As one participant explained: *“Learning to make Kaingang crafts is also learning about our history and the values of our culture”* (S1).

**Figure 1.**
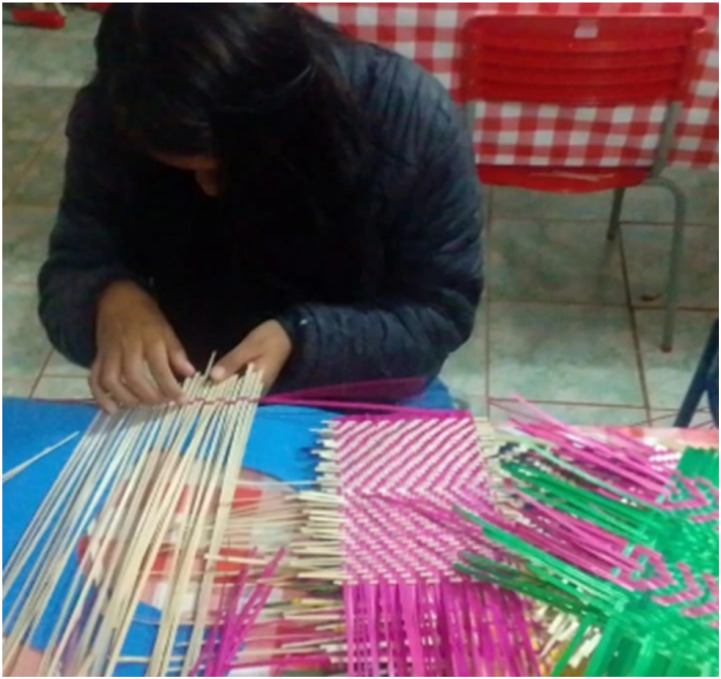
Handcraft making

**Figure 2.**
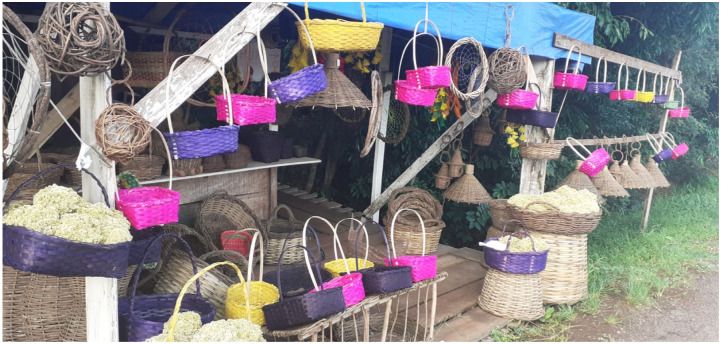
Exhibition of handicrafts for sale

**Figure 3.**
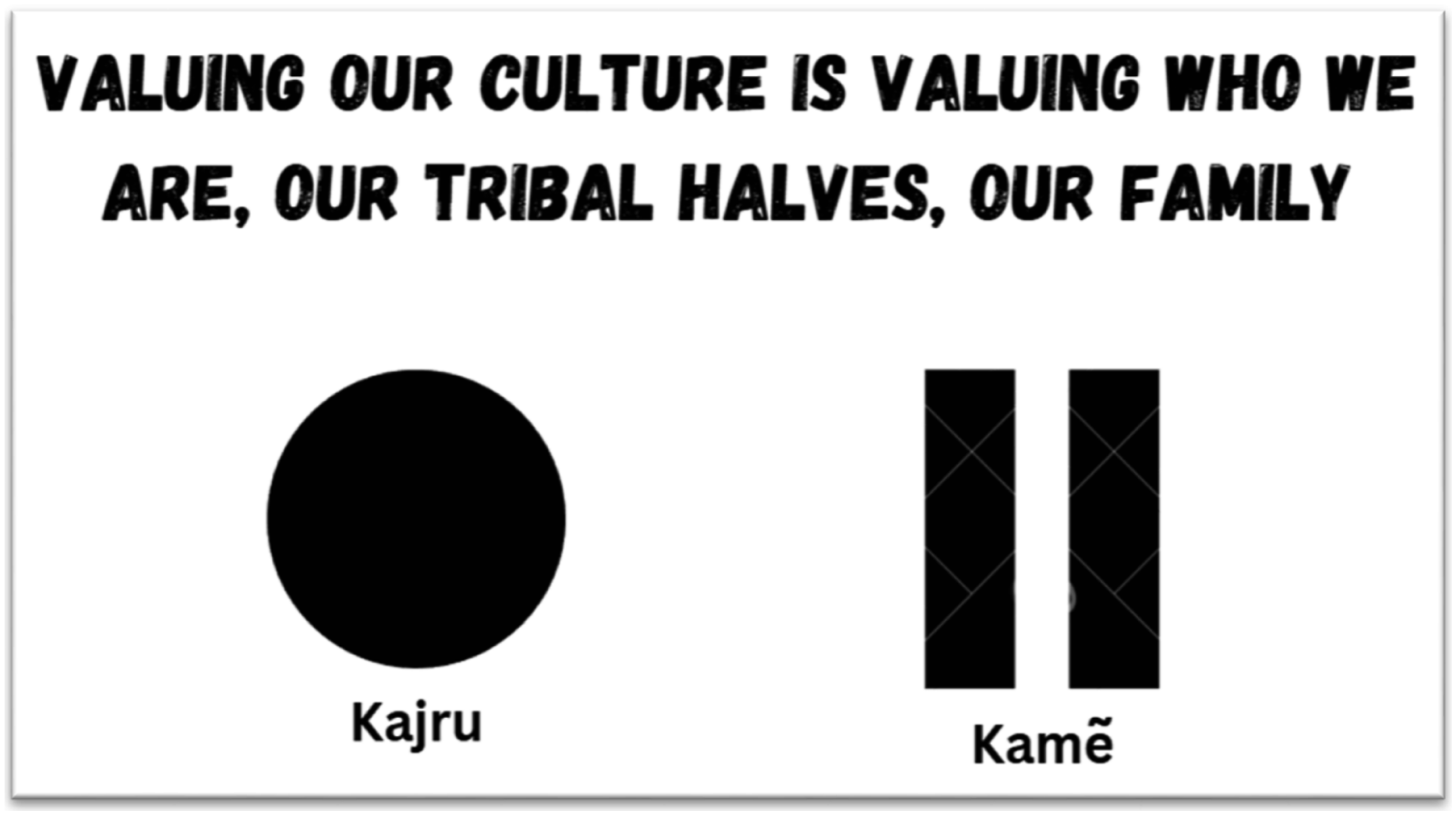
Graphic representations that represent the *Kajru* and *Kamẽ brands*

**Figure 4.**
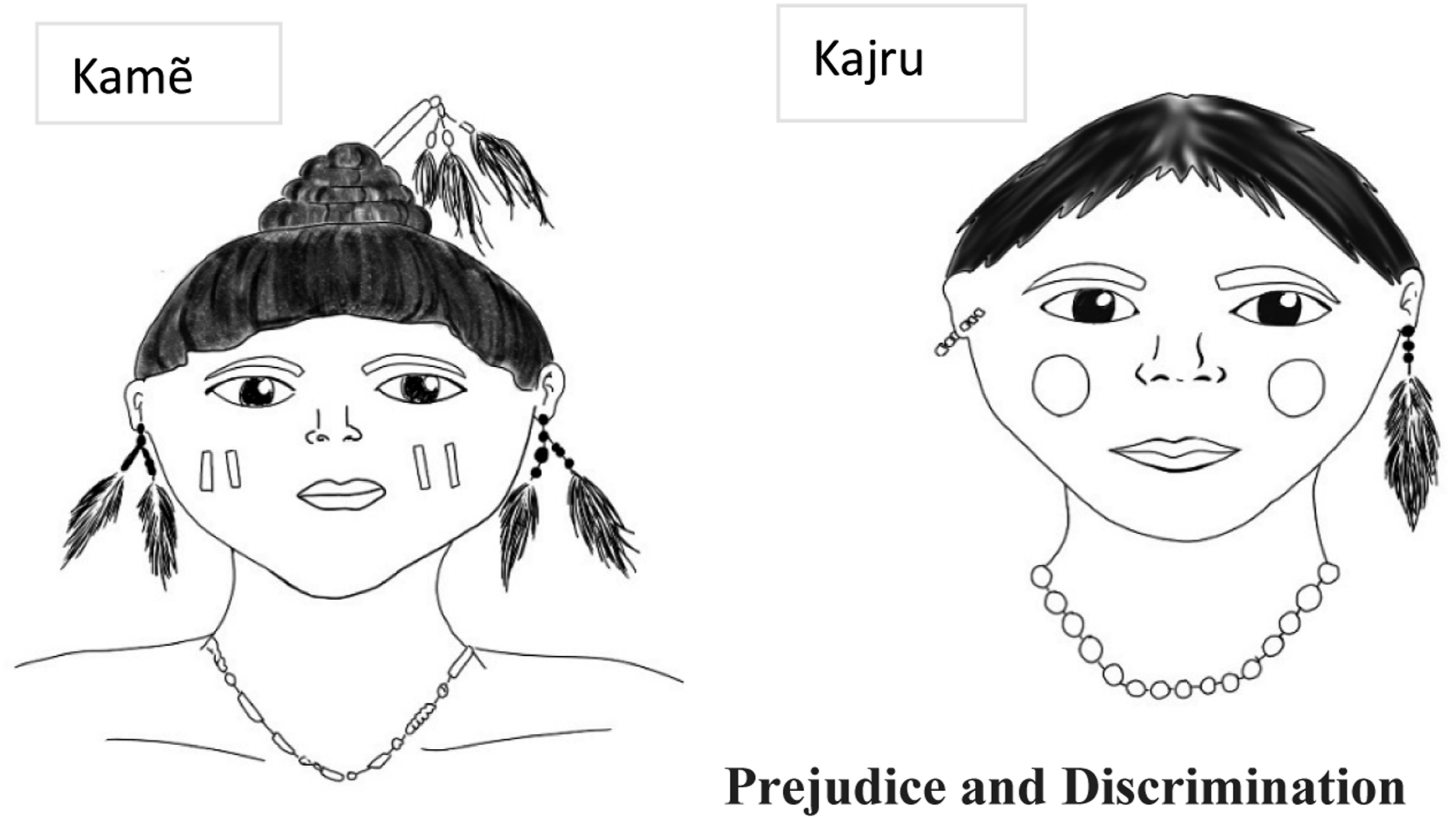
Representation of a Kamẽ girl and of a *Kajru* boy. (A) Kamẽ. (B) Kajru

**Figure 5.**
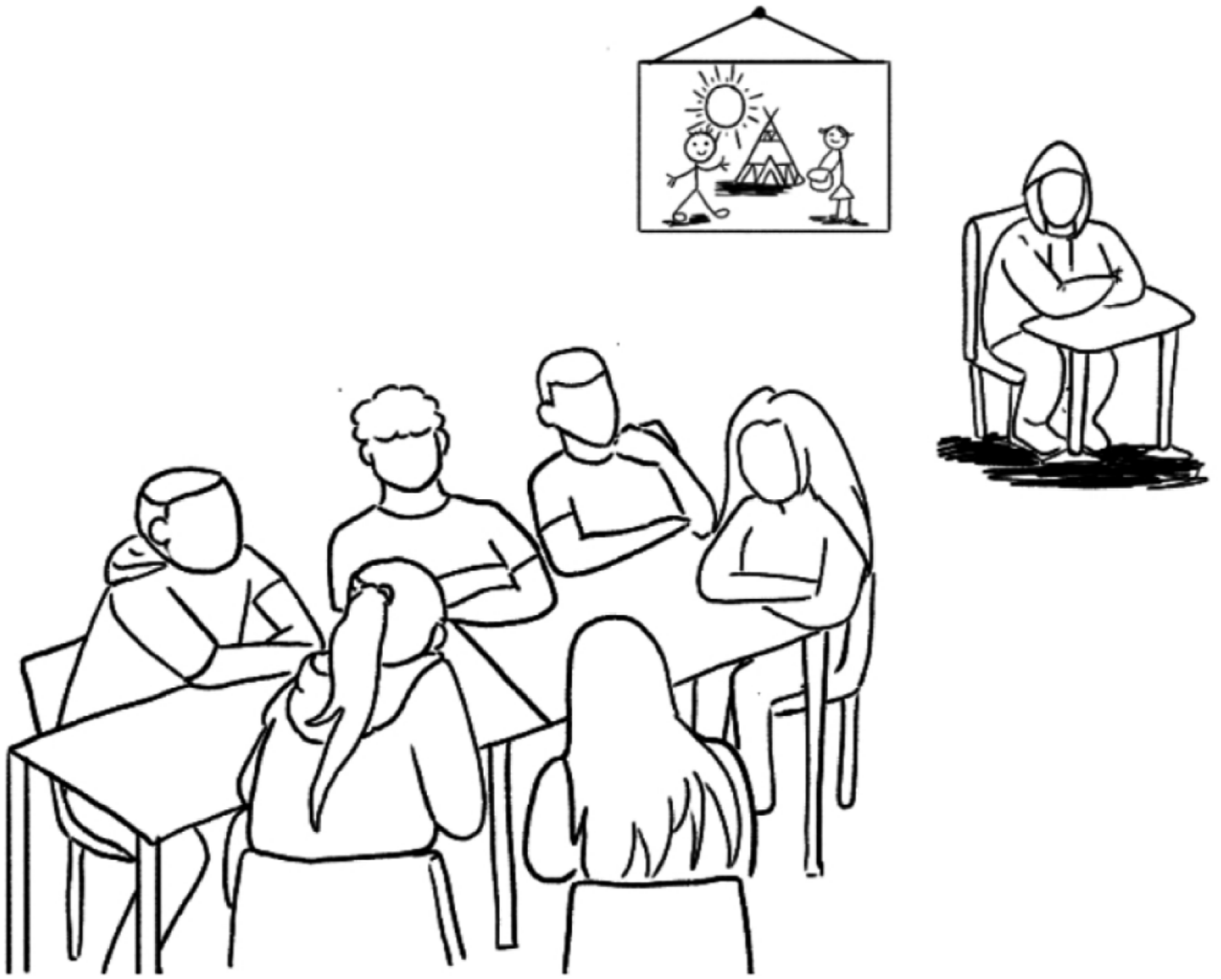
Representation of exclusion among students

**Figure 6.**
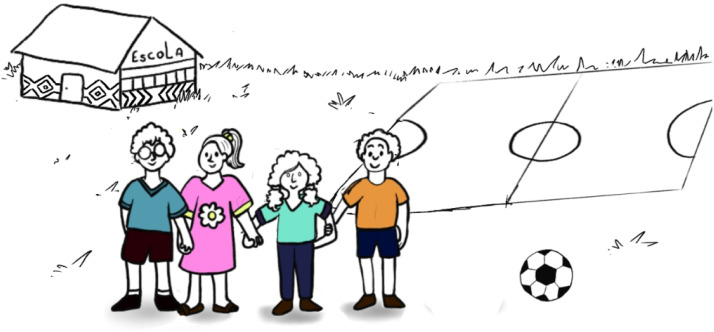
Representation of inclusion and respect for differences

**Figure 7.**
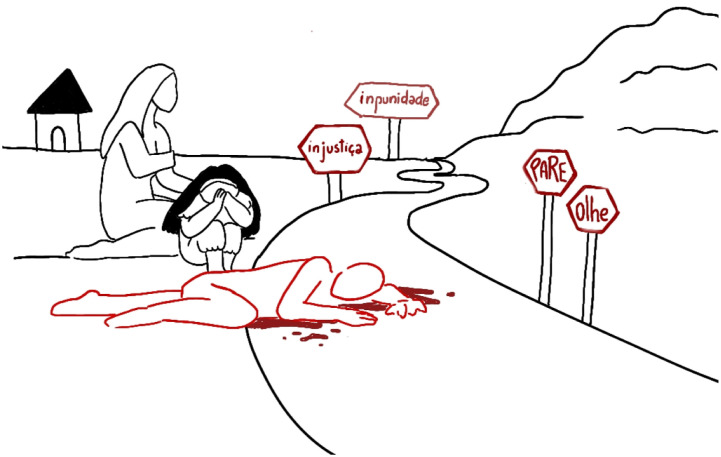
Representation of violence and its implications

**Figure 8.**
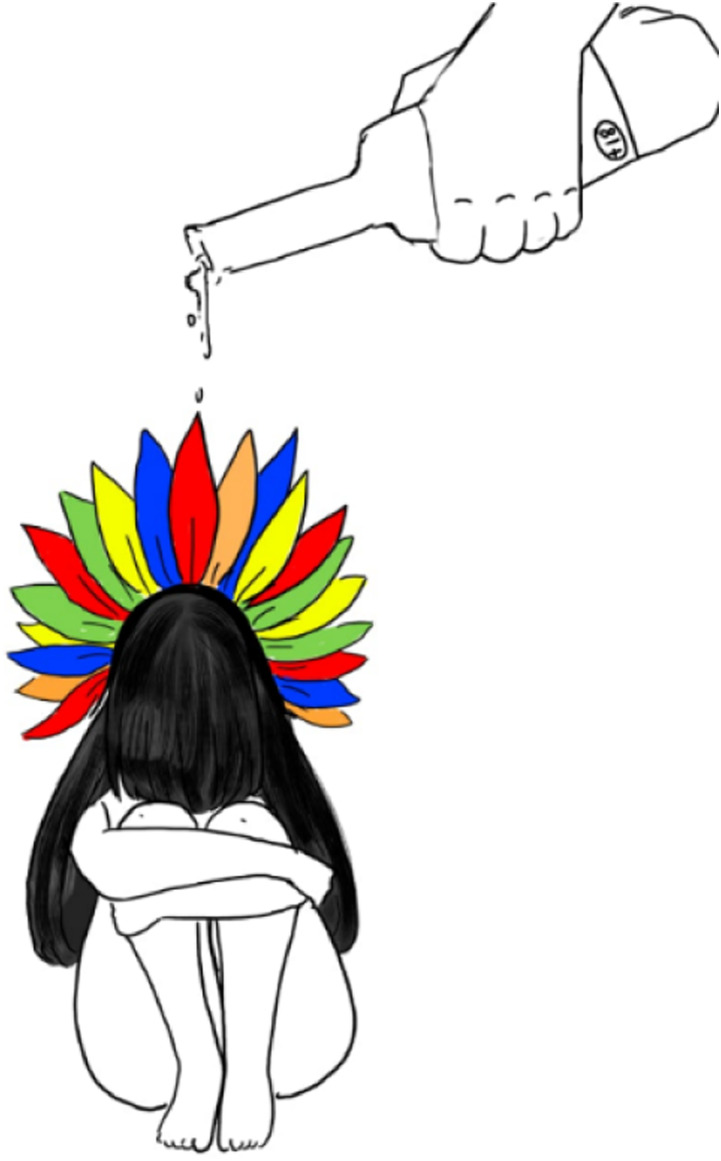
Representation of the disorders caused by alcohol use

Vignettes consistently featured the symbolic importance of Kaingang moiety markers—“Kajru” and “Kamẽ”—represented through circles and parallel lines inherited patrilineally and expressed in graphic arts, body painting, clothing, and crafts (Figures 1-8). Participants articulated the complementary nature of these divisions: *“The Kajru is different from the Kamẽ, but they complement each other and form the Kaingang people”* (S2).

### Marriage Traditions and Contemporary Challenges

Traditional marriage practices between different moieties were prominently featured, with participants recognizing this system as essential for cultural maintenance. However, vignettes also revealed contemporary tensions arising from increased interethnic marriages. Participants expressed concerns about identity negotiation among children of mixed heritage, as illustrated by one participant’s experience:

*“My father is indigenous and my mother is not. I live in the territory and feel indigenous, but some of my colleagues say that I am not indigenous, they call me mixed race and say that I am not like them. When I go to the city to visit my relatives who are not indigenous, I am also treated differently by them”* (S3).

## Environmental Degradation and Cultural Disruption

Environmental themes appeared prominently across vignettes, with participants linking ecological destruction to cultural preservation challenges. Deforestation, river pollution, and resource scarcity were identified as immediate threats to traditional practices. One participant noted: *“It is increasingly difficult to find materials to make crafts, everything is being deforested to make way for crops”* (S6).

Environmental concerns extended beyond material impacts to encompass spiritual and cultural dimensions of territorial relationships. A 12-year-old participant adapted the song “Planeta Azul” to express these connections: “Life and nature are always at the mercy of pollution. The seasons are reversed, it is hot in winter and cold in summer. Fish are dying in the rivers. Animal species are becoming extinct” (Male, age 12, Session 9).

## External Discrimination

Participants consistently reported experiencing discrimination in neighbouring urban areas, particularly when selling crafts or conducting other activities outside the territory. The pejorative term “bugre” was frequently encountered, causing significant emotional distress. Representative experiences included:

*“I feel sad when I hear people say, ‘Give these bugres a loaf of bread to get them out of the front of my house’”* (S4)

*“One day we were in the city and they said they were going to give us lunch, they put us on a bus and took us, but there was no lunch, they just left us on the highway because they didn't want us in the city”* (S5)

## Internal Community Tensions

While external discrimination was more intense, participants also acknowledged prejudice within the community, particularly affecting individuals of mixed heritage or those perceived as culturally different. These experiences led to social isolation and exclusion, as depicted in multiple vignettes showing students separated from peer groups.

## Violence and Substance Use

Violence emerged as a persistent concern, reflecting ongoing conflicts within Guarita Indigenous Territory. Participants noted three youth murders in early 2025 alone, contributing to community-wide trauma and insecurity. Vignettes consistently linked violence to alcohol and substance use, which participants identified as frequent triggers for family and community conflicts.

Participants expressed alarm at increasing substance use among younger adolescents, recognizing this trend as both symptom and catalyst of broader community distress. These themes appeared across multiple artistic formats, demonstrating the pervasive impact on daily life.

## Social Integration and Cultural Resistance

### Positive Coping Strategies

Despite documenting significant challenges, vignettes also highlighted resilience and resistance strategies. Participants created content promoting inclusion, respect for differences, and community solidarity. A notable example was a parody of “Trem Bala” emphasizing friendship and mutual support:

*“It’s not about pushing everyone at school away from you / It’s about knowing that someone needs you somewhere / Lean on your friend's shoulder, smile, hug the classmates around you / It's about knowing how to be a friend to your friend and your teacher”* (Female, age 12, Session 9).

### Sports and Collective Activities

Collective games, particularly soccer, were consistently identified as effective integration strategies promoting belonging and social cohesion. Video vignettes showed how teacher-facilitated inclusion activities could successfully integrate marginalized students into peer groups.

### Cultural Revival Initiatives

Participants emphasized cultural revival as active resistance, highlighting traditional ceremonies, medicinal plant knowledge, and intergenerational transmission as protective factors for collective wellbeing. The programme itself catalyzed cultural revival activities, with teachers initiating ancestral dance rehearsals and traditional costume production for school and community cultural events.

### Protective and Risk Factors

Analysis across all vignettes revealed consistent patterns of protective factors including family support, cultural practice engagement, sports participation, and strong community connections. Risk factors encompassed discrimination experiences, environmental degradation, substance use, violence exposure, and cultural disconnection. These findings demonstrate the complex interplay between individual, family, community, and structural factors influencing Indigenous adolescent mental health.

## Discussion

### Vignettes as Culturally Responsive Mental Health Tools

This study demonstrates that adolescent-created vignettes represent a powerful, culturally responsive methodology for Indigenous mental health promotion that extends far beyond traditional expression tools.^16-18^ Our findings reveal that vignettes function as sophisticated mechanisms for listening, meaning-making, and knowledge production^18-20^ while strengthening adolescent autonomy and agency within their cultural contexts. These creative narratives served as symbolic bridges connecting lived experiences with reflective dialogue, enabling exploration of complex topics including violence, social exclusion, and identity negotiation without compromising individual privacy or violating cultural protocols around personal disclosure. The vignettes inform the IMPACT programme sessions.

The perspectives about Indigenous context relates to collective dynamics of the community. Unlike direct personal disclosure methods common in Western therapeutic approaches, vignettes allowed participants to address sensitive topics through symbolic representation, maintaining cultural boundaries while facilitating meaningful dialogue.^21^ This approach proved particularly valuable for discussing psychological distress, family conflicts, and identity struggles—topics that might otherwise remain unexpressed due to cultural norms or fear of stigmatization.

The vignettes produced through this process also have practical applicability beyond the immediate study context. They may serve as pedagogical and dialogical tools within schools, primary care settings, and community initiatives, facilitating conversations around mental health in ways that are culturally resonant and non-stigmatizing. While these materials were developed by adolescents not currently identified as experiencing severe mental health problems, their symbolic and narrative structure allows for adaptation across different levels of need. However, their use with adolescents experiencing significant psychological distress should be approached with contextual sensitivity, and future research is needed to evaluate their perceived impact and appropriateness within more clinically vulnerable populations.

Importantly, although the participating adolescents were selected based on school engagement and did not necessarily represent those experiencing the most severe psychological distress, their contributions remain highly relevant for mental health promotion. From a preventive perspective, adolescents who are more socially and academically engaged are often key agents in identifying and reinforcing protective factors within their communities. The vignettes developed in this study illuminate culturally grounded sources of resilience—including family cohesion, cultural identity, spirituality, and collective activities—which are central to Indigenous conceptualizations of wellbeing. As such, these narratives contribute to upstream mental health promotion by fostering shared understanding, strengthening positive norms, and creating culturally safe spaces for dialogue that may indirectly benefit more vulnerable youth.

### Participatory Methodology and Youth Agency

The collaborative vignette creation processes exemplified genuine participatory research principles by positioning adolescents as knowledge producers rather than passive research subjects.^17,19^ This methodological approach aligns with Indigenous research paradigms emphasizing community control over knowledge production and the recognition of Indigenous youth as cultural experts regarding their own experiences. The authenticity of participant-generated content contributed to more representative data aligned with adolescent-attributed meanings, facilitating genuine listening and enhanced communication power between researchers and community members.^17^

The vignettes’ grounding in concrete experiences enhanced their pedagogical value while affirming intercultural dialogue and youth protagonism. This process enabled early identification of psychosocial risk factors while simultaneously strengthening protective factors through mediated conversations about conflicts, emotions, relationships, and identity formation.^22,23^ The methodology thus facilitated both individual expression and collective reflection, broadening understanding of challenges facing Indigenous adolescents while building community capacity for ongoing mental health support.

## Complex Mental Health Landscape

### Structural Violence and Cultural Disruption

Our findings reveal a complex mental health landscape shaped by intersecting structural, cultural, and environmental factors. Participants’ accounts documented serious violence including physical assault, suicide, homicide, and retaliatory acts—experiences that reflect broader patterns of structural violence affecting Indigenous communities.^4,5^ The intensification of contact with Western culture, persistent social inequalities, and increasing interethnic relationships emerged as contributing factors to prejudice and discrimination, highlighting the need for care strategies addressing both structural determinants and emotional-symbolic dimensions of Indigenous adolescent wellbeing.^8^

Environmental degradation—including deforestation, river pollution, and resource scarcity—represented not merely ecological concerns but fundamental disruptions to cultural and spiritual connections with territory. These findings align with growing recognition that environmental health and Indigenous mental health are inextricably linked, with territorial degradation constituting a form of cultural violence that undermines traditional knowledge systems and spiritual practices essential for community wellbeing.^24^

These findings must also be interpreted within the broader context of colonialism and its enduring impacts on Indigenous communities. Historical processes of territorial dispossession, cultural suppression, and forced assimilation continue to shape contemporary experiences of marginalization and psychological distress. Intergenerational trauma, transmitted through disrupted cultural practices and collective memory, further compounds these challenges. In this context, environmental degradation is not solely an ecological issue but a direct threat to land-based identity, spirituality, and cultural continuity—dimensions that are inseparable from mental health in Indigenous worldviews. Recognizing these structural determinants is essential for avoiding individualizing interpretations of distress and for informing culturally grounded and socially just mental health programmes.

### Cultural Revival as Resistance and Healing

Conversely, participants consistently identified cultural revival and ancestral value recovery as pathways to empowerment and healing.^23,25^ Traditional practices including craftsmanship, medicinal plant knowledge, and intergenerational learning emerged as protective factors fostering reconnection and collective belonging. These cultural productions function as mobilizing forces across school, family, and community contexts, contributing significantly to health promotion and wellbeing maintenance.

The prominence of sports as social integration mechanisms further demonstrates how culturally relevant activities can bridge traditional and contemporary contexts while fostering community cohesion. These findings support theoretical frameworks positioning cultural continuity as a fundamental protective factor for Indigenous mental health, with cultural practice engagement serving both individual and collective healing functions.^25^

### Educational Contexts as Sites of Tension and Opportunity

Schools emerged as ambiguous spaces embodying both opportunity and challenge for Indigenous adolescents. While educational settings provided venues for cultural identity strengthening, peer connection, and learning, they simultaneously represented sites of discrimination, cultural tension, and identity negotiation struggles. These experiences reflect broader patterns documented among Indigenous adolescents across diverse contexts, suggesting systemic rather than localized challenges.^16,26^

This educational ambiguity underscores the critical need for intercultural approaches that actively incorporate Indigenous worldviews, knowledge systems, and pedagogical practices rather than merely accommodating cultural differences within Western educational frameworks. Effective educational programmes must create safe spaces for identity affirmation while addressing discrimination and promoting inclusive practices that value cultural diversity as educational assets rather than obstacles.

### Implications for Intercultural Mental Health Practice

Our findings challenge conventional cultural competency approaches by demonstrating the necessity of genuine intercultural practice that positions Indigenous knowledge systems as equal to Western frameworks rather than supplementary considerations.^18-20^ Effective mental health promotion in Indigenous contexts requires recognizing adolescents as active agents in solution construction rather than passive care recipients,^17^ supporting community-controlled programmes that honor Indigenous sovereignty over health and wellbeing approaches.

While this study demonstrates the potential of community-based creative programmes, findings also highlight the necessity of addressing structural determinants of Indigenous mental health including discrimination, environmental degradation, violence, and educational inequities.^4,5,8^ Effective mental health promotion requires multi-level programmes combining community-controlled cultural programs with advocacy for policy changes addressing systemic marginalization and rights violations.

### Study Limitations and Future Directions

This study’s limitations include its single-site design and relatively small sample size, which may limit generalizability across diverse Indigenous contexts. A further and important limitation concerns the recruitment criteria, which required positive academic standing and consistent school engagement. This approach likely produced a sample skewed toward adolescents who are better integrated into institutional settings, thereby excluding youth who are truant, disengaged, or experiencing more severe psychological distress—the very population that may be at heightened psychosocial risk. As a result, the vignettes and discussions may not fully capture the perspectives and mental health experiences of the most vulnerable Kaingang adolescents. Future studies should deliberately seek to include youth with lower school attendance or engagement, potentially through alternative recruitment pathways such as community centers, youth justice settings, or informal community networks.

Future research should also examine vignette-based programmes across multiple Indigenous communities to assess cultural transferability and adaptation requirements. Longitudinal studies are needed to evaluate sustained impacts of participatory creative programmes on adolescent mental health outcomes and community capacity building. Despite these limitations, our findings provide compelling evidence for the effectiveness of culturally grounded, youth-led creative programmes in Indigenous mental health promotion.

This sampling approach may also limit the transferability of findings to adolescents experiencing greater psychosocial vulnerability, including those disengaged from school or facing more severe mental health challenges. As such, the findings should be interpreted primarily as reflecting the perspectives of more engaged youth, who may possess distinct protective profiles. Future research should prioritize inclusive recruitment strategies to better capture the full spectrum of adolescent experiences within Indigenous communities.

## Conclusion

This study demonstrates that collaborative vignette creation represents a culturally responsive and methodologically sound approach to Indigenous adolescent mental health promotion. Among Kaingang youth in Guarita Indigenous Territory, vignettes functioned as sophisticated tools extending beyond simple expression to serve as catalysts for collective reflection, identity strengthening, and community bond building. The methodology successfully created safe spaces for dialogue around psychological wellbeing topics while honoring cultural protocols and values essential to Indigenous communities.

The participatory vignette creation process proved effective in centering adolescent voice while respecting Indigenous knowledge systems and communication practices. Unlike conventional mental health programmes, which often impose Western therapeutic frameworks, this approach emerged organically from community needs and cultural strengths, positioning youth as knowledge producers and solution architects rather than passive recipients of a programme.

Findings reveal that when provided with culturally appropriate platforms for expression, Indigenous adolescents demonstrate remarkable capacity for articulating complex mental health experiences, identifying community strengths and challenges, and generating creative solutions to psychological distress. It is nonetheless important to note that the adolescents who participated were selected partly on the basis of school engagement; thus, the insights gained, while rich, may not fully represent the breadth of experiences across the Kaingang adolescent population, particularly those facing the greatest adversity. The vignettes facilitated both individual healing and collective processing of shared traumas including discrimination, violence, and cultural disruption while simultaneously celebrating cultural continuity and resilience.

This research contributes to growing evidence that effective Indigenous mental health promotion requires methodological innovation grounded in community-controlled, culturally responsive approaches. As Indigenous communities continue to navigate complex challenges including cultural disruption, environmental degradation, and systemic discrimination, methodologies that honor youth agency and cultural wisdom become increasingly essential. In summary, Indigenous communities led the development of an innovative cultural tool (art-based vignettes) to promote positive adolescent mental health in the IMPACT programme.

## Data Availability

The datasets generated and/or analyzed during the current study are not publicly available due to ethical restrictions involving Indigenous community confidentiality but are available from the corresponding author on reasonable request and with approval from Indigenous leadership of the Guarita Territory.*
